# Report of Committee Appointed to Visit Members of A. S. D. S., in New York

**Published:** 1846-03

**Authors:** 


*Report of Committee appointed to visit Members of A. S. D. S. in New York.*—We are requested to state that when this committee called on A. W. Brown, Dentist, of Park Place, that he stated that he *never had used a particle of amalgam in any way for dental purposes, and never would;* but did not wish to sign a pledge not to do so. In the report, as published, Mr. B. is represented as stating that he was in the habit of using it, and as objecting to sign a pledge not to do so. We regret this error in the report of the committee, and are sure it was unintentional on their part.

